# YAPPIS-Finder: A novel method for protein-protein interaction site predictions

**DOI:** 10.6026/97320630018604

**Published:** 2022-07-31

**Authors:** Vicky Kumar, Ashita Sood, Anjana Munshi, Tarkeshwar Gautam, Mahesh Kulharia

**Affiliations:** 1 Centre for Computational Sciences, Central University of Punjab, Bathinda, Punjab, India; 2 Centre for Computational Biology and Bioinformatics, Central University of Himachal Pradesh, Dharamsala, Himachal Pradesh, India; 3 Department of Human Genetics and Molecular Medicine, School of Health Sciences, Central University of Punjab, Bathinda, India; 4 Department of Zoology, Kalindi College, University of Delhi, Delhi, India

**Keywords:** YAPPIS-Finder, protein-protein interaction site, predictions

## Abstract

We describe a multi parametric-approach, YAPPIS-Finder, for predicting the PPI sites on protein surface. A non-redundant database of comprised of 2,265 protein-protein interaction interfaces (PPIIs) involving 4,530 protein-protein interacting partners (PPIPs) and depicting the interaction between protein-chains of experimentally determined PPCs was used in designing the YAPPIS-Finder. Parametric score obtained on analyzing these 4,530 PPIPs with respect to their residue interface propensity, their hydrophobic content, and amount of solvation free energy associated with them provided the basis of YAPPIS-Finder. By applying YAPPIS-Finder on another dataset 4,290 PPIPs from 2,145 PPIIs, the optimal range of the parametric scores and protein-probe van der Waals energy of interaction was determined. Subsequently, taking the optimal range of PPIP parametric scores and threshold for protein-probe van der Waals energy of interaction into the consideration, the YAPPIS-Finder was tested on a blind dataset of 554 protein-chains and it was found predicting 69.67% sites correctly. On predicting only one PPI site on each protein-chain, the YAPPIS-Finder found covering 22.91% of actually sites in the predicted site. Contrary to this, the sites predicted by SPPIDER covered 22.7% of actual sites. However, on predicting two PPI sites for each protein-chain, the percentage coverage of actual sites in the predicted sites by YAPPIS-Finder exceeded two-fold (i.e. 41.81%), thus making the YAPPIS-Finder a better method.

## Background:

Proteins are the basic functional unit in cellular world of life [1]. They are genetically programmed to enact an array of molecular functions in response to biological events at cellular and system levels [2,3]. The unquestionable roles that proteins play in executing various intra- and extra-cellular processes such as cell proliferation, differentiation, apoptosis, and signal transduction have drawn the attention of the scientific community to get their structural and functional insights. In various studies, the efficacy of proteins in the cellular environment is reported to be of short-range and inadequate to sustain life in isolation [4]. More often, proteins interactively associate with other bio-molecules to form supramolecular assemblies responsible for molecular functioning in living organisms. Understanding the molecular phenomenon that triggers and maintains the complexity of such associations may improve the application of protein chemistry. Formation of protein-protein complexes (PPCs) are the outcome of one of such molecular association in which the interaction between two proteins is governed by formation of covalent and non-covalent associations between them [5, 6]. The covalent PPIs, although rare to see, are owed to sharing of electrons between the protein constituents [7]. While in the case of non-covalent interactions, formation of hydrogen bonds, ionic interactions, van der Waals interactions, or hydrophobic bonds, are the main contributing factors for proteins complexation [8]. The presence of such binding factors help in associating two proteins, thereby, assists in maintenance of life. Therefore, development of an approach emphasizing on the protein dynamics in context of preference of binding partners, binding site location, functionality concomitant with the formed protein complexes, is the need of hour. Despite the availability of experimental techniques for identification of PPIs in abundance [9] [10] [11] recognition of binding sites for proteins like Wnt [12, 13] (or similar hydrophobic protein) and Hedgehog [14] (membrane proteins), even though very much crucial, is difficult. Additionally, relatively high experimental cost and time-intensiveness nature of experimental techniques makes them less apt for application en masse. Therefore, development and application of computational methods, which are free from such problems, are growing rapidly. In the proposed work, we have presented an approach named Yet Another Protein Protein Interaction Site-Finder (YAPPIS-Finder) for predicting the PPI sites on protein surface. A non-redundant database of comprised of 2,265 PPIIs (or 4,530 PPIPs) depicting the interaction sites between two protein-chains of experimentally determined PPCs was used in designing YAPPIS-Finder. Analysis of these 4,530 PPIPs was carried out to understand the PPI sites with respect to their residue interface propensity, their hydrophobic content, and amount of solvation free energy associated with them. Another dataset of 2,145 PPIIs (4,290 PPIPs) was used to train the proposed approach and subsequently the optimal range of PPIPs parametric scores was derived. The approach was tested on a blind dataset of 554 protein-chains and its performance was also compared with the SPPIDER. [15]

## Materials and Methods:

A non-redundant database protein-protein interaction interfaces (NRDB)[16] depicting the actual PPI sites was analyzed with respect to residue interface propensity (RIP), hydrophobicity and solvation free energy to design the proposed computational approach for PPI sites prediction.

### Non-redundant database of protein-protein interaction interfaces (NRDB) depicting actual interfaces:

A non-redundant database of PPIIs 17] demarcated from experimentally determined PPCs was used in proposed study. NRDB was designed considering the PDBs for which the information of structural classification of protein was available in the last manually curated SCOP version 1.75. In NRDB, the information of interacting interface was determined by considering the interatomic distance between the constituting atoms of two protein-chains in a PPC. Two atoms belonging to two different protein-chains of a PPC were said to be in contact and demarcated as an atomic contact pair (ACP) if the intervening distance between them was less than the sum of their van der Waals radii plus 1 Å as tolerance factor (Figure 1 - see PDF). The collection of ACPs between a pair of interacting protein-chains was referred as "Protein-Protein Interaction Interface" (PPII) and the collection of interacting atoms from individual interacting protein-chain were termed as the "Protein-Protein Interacting Patch" (PPIP). Only the PPIIs with at least 20 ACPs were retained in the NRDB and a total of 2,265 PPIIs (4,530 PPIPs) from 1,931 PDB files were demarcated. These 2,265 PPIIs were representing 43,509 PPIIs and the same number of SCOP super family pairs.

### Analysis of PPIPs with respect to residue interface propensity (RIP):

All of the PPIPs from NRDB 16] were examined with respect to RIP and the cumulative RIP score for each PPIP was carried out by taking into the account the RIP scores of individual residues derived in our unpublished work. For each interacting atom in a PPIP, its individual RIP score was calculated by dividing the overall RIP score of the residue by number of atoms the residue, whose this interacting atoms is a part, does normally have (ignoring the atoms involved in peptide bond formation). This was followed by the summation of individual RIP score of all interacting atoms in the PPII to represent the cumulative RIP score of the PPIP (Eq. 1 - see PDF).

### Analysis of PPIPs with respect to hydrophobicity:

To determine the level of hydrophobicity (ϕ) associated with PPIPs, the hydrophobicity scale for amino acids given by Hessa et al. 2005 was used [18]. For each interacting atom in the PPIP, its corresponding hydrophobicity score was obtained by dividing the overall residue hydrophobicity score by number of atoms the residue, whose this interacting atom is a part, does normally have (ignoring the atoms involved in peptide bond formation). At last, the hydrophobicity score of all interacting atoms in the PPIP was summed up in linear fashion to represent the hydrophobicity score of the PPIP (Eq. 2 - see PDF)

### Analysis of PPIPs with respect to solvation free energy:

The solvation free energy of PPIP was calculated by taking into account the solvation energy scale for amino acids given by [19] White et al, 1996 for each interacting atom in PPIP, its corresponding solvation free energy score was calculated by dividing the overall solvation free energy of the residue by number of atoms the residue, whose this interacting atoms is a part, does normally have (ignoring the atoms involved in peptide bond formation). This was followed by the summation of solvation free energy score of all interacting atoms in the PPII to represent the solvation energy score of the PPIP (Eq. 3 - see PDF).

### Removal of outliers from the parametric scores of PPIPs and decomposition of parametric scores into sub-ranges:

The parametric scores obtained on analyzing the PPIPs from NRDB were set to provide foundation for the proposed scheme. However, the NRDB analysis revealed that the parametric scores for PPIPs contained a significant number of outliers in them. Therefore, a statistical approach of inter quartile range was adopted to remove the outliers. After outlier removal, the entire range of parametric score for each PPI sites parameter was divided into a number of bins with width calculated using Scott’'s rule (Eq. 4 - see PDF).

### Creation of the training and test dataset to implement the proposed approach:

Success of any prediction tool largely depends of quality of the datasets used in its designing. The dataset should be comprised of information related to both known interacting protein pairs (positive set) and non-interacting protein pairs (negative set). It is quite easy to obtain the experimental instances of PPI for the positive set while construction of negative set is not that much straightforward. In the proposed study, to develop a computational approach for PPI prediction, one training and one test set was designed considering the presence or absence of SCOP superfamily pair in NRDB proposed by our research group previously 17]. To design the training set, the SCOP 20, 21] superfamily pairs with their corresponding PPIIs in the NRDB were taken into the account. However, to remove the overlap between the NRDB and training set, for each SCOP 20, 21] superfamily pair covered in NRDB, a new PPII representative was selected from PPInS 17]. If there were more than one PPII available for a SCOP superfamily pair in PPInS, then the PPII with the largest number of ACPs was selected. If the PPII with the largest number of ACPs was the one which was already a part of NRDB 16] , then the PPII with second largest number of ACPs was selected for the training set. Following this strategy, a total of 2,145 PPIIs from 1,896 PDBs were selected as training set. To design the test set, the SCOP superfamily pairs which were not covered in NRDB 16] were selected. For each such SCOP superfamily pair, the PPII with the largest number of ACPs was selected from PPInS as a part of the test set. In this way, no room was left for overlap between the training and test set and a total of 554 binary PPIIs from 277 PDBs were selected as test set.

### Implementation of YAPPIS-Finder:

#### (i) Overview:

The implementation of the proposed computational approach was completed in two phases; training and testing phase. During training phase, initially, using all of the PPIIs involved in NRDB [16] , the optimal range of PPI sites' parametric scores was calculated. The obtained ranges were then decomposed into multiple sub-ranges (called domains) using the method of interquartile range. This was followed by examining the surface of unbounded proteins extracted from the experimentally determined PPCs contained in training dataset. Taking two parameters at a time and their corresponding domain scores one at a time along with the threshold for protein-probe van der Waals energy of interaction, the protein surface was examined using an energy-based grid-oriented approach. The group of atoms with parametric scores within the range of current domain and protein-probe van der Waals interaction energy threshold were clustered into 15 clusters (created considering the spatial proximity of predicted interacting atoms). The obtained clusters were recognized as possible PPI sites and a predicted site score (Ƭ) representing the precision and coverage of predicted PPI sites against the actual sites was also calculated for each of them.

This was followed by the identification of the parametric scores and protein-probe interaction energy conforming Ƭ≥0.25(25%) against the actual PPI sites (demarcated on the basis ACP definition). The obtained parametric scores and threshold for protein-probe van der Waals interaction energy were designated as the optimal range of the parameters. This way, the optimal ranges of all of the parameters were calculated.

During the testing phase, the unbound forms of protein-chains extracted from the experimentally determined PPCs contained in testing dataset were examined using the similar strategy. The protein surface was examined corresponding to the optimal ranges of PPI sites parametric score and the optimal value of the van der Waals energy of interaction obtained during the training phase. For each PPI site parameter pair, the protein atoms with their parametric scores within the threshold values (i.e. optimal range) were clustered on the basis of geometric proximity and ranked using Eq. 5-7(see PDF). Here too, only the predicted sites with Ƭ≥0.25(25%) were termed as the correctly predicted sites. Following this, the best ranked predicted sites were compared against the actual PPI sites to evaluate the prediction efficacy.

### YAPPIS-Finder algorithm:

#### Input:

Protein-chains extracted from experimentally determined protein-protein complex.

#### Process:

The input protein-chain was placed in a three-dimensional box, which was divided into a cubic grid of resolution 0.9 Å. At each grid point, a methyl (-CH3) probe was placed and van der Waals energy of interaction was calculated between the protein atom and the methyl probe. The process of calculating the protein-probe van der Waals interaction energy is described in detail in Laurie and Jackson [22]. The interaction energy was calculated using the GRID force field parameters as described in 23]. Grid points with a "protein–probe interaction" energy more favourable (negative) than a predetermined threshold were retained. For such grid points, three protein binding propensity scores (i.e. residue interface propensity score, solvation energy propensity score, and hydrophobicity propensity score) were calculated by considering the type of amino acid residue whose atoms are occluded from solvent exposure due to the predicted grid points. An amino acid is considered to be interacting with a grid point if at least one of its atoms is within 1.6 Å of the grid point. The overall protein binding propensity of the grid point, k, was defined as:(see PDF)

#### Output:

Clusters of atoms as the predicted PPI sites.

#### Comparison of prediction power of SPPIDER and YAPPIS-Finder:

The prediction power of YAPPIS-Finder was also compared against the SPPIDER [15] which is one of most efficient approach (as claimed by its designer) available. The protein-chains from the test dataset were examined using SPPIDER to predict the PPI sites and prediction power of both these approaches, SPPIDER 15] and YAPPIS-Finder, was compared using Eq. 5-7(see PDF).

## Results and Discussion:

Optimization of protein-protein interaction sites parameters by running YAPPIS-Finder for the training dataset

To obtain the optimal range of PPI site parameters, the YAPPIS-Finder algorithm was applied on protein chains extracted from experimentally determined PPCs of training dataset as described in Step 4 and Figure 3(see PDF). The predicted and actual sites were compared (demarcated using the definition of ACP) and overlap between the two was determined using Eq. 8 to 10(see PDF). Subsequently, the values of parametric scores and threshold of van der Waals energy for interaction were retrieved for which,Ƭ≥0.25(25%). The parametric scores obtained were termed as the optimistic range of the parameters under consideration.

Prediction of interaction sites for the test dataset proteins by YAPPIS-Finder and performance analysis was done in similar manner.

Only predicted sites with Ƭ≥0.25(25%) were termed as the correctly predicted sites.

Prediction efficacy of YAPPIS-Finder and SPPIDER and comparison of their performance evaluation The prediction efficacy of YAPPIS-Finder was examined by running it on a blind dataset of 554 protein-chains from the test set. For these 554 protein-chains, their actual PPI sites were demarcated using the definition of ACP described in Materials and Methods section this section as well as in [17]. Using YAPPIS-Finder with optimistic range of PPIP parametric scores and optimal values of protein-probe van der Waals energy of interaction, 10 PPI sites were predicted for each protein-chain. However, to minimize the false negatives, the sites with Ƭ≥0.25(25%) were termed as the correctly predicted sites. This way, a total of 385 sites were termed as the correctly predicted against the 554 actual sites giving us the prediction accuracy of 69.67%. When all these 554 protein-chains were given to SPPIDER server [15] for site prediction, total 529 sites were predicted by it. However, opposite to your approach where we have put filtering criteria of Ƭ≥0.25(25%) for a site to be considered as the correctly predicted site, the SPPIDER has even predicted the site with only one residue, which may be a result of false prediction as well.

Comparison between the performance evaluation of YAPPIS-Finder and SPPIDER

Following to the determination of prediction efficacy of the YAPPIS-Finder and SPPIDER revealed that SPPIDER, the sites predicted by both of these approaches were compared against the actual sites. On predicting only one PPI site for each protein-chain, the YAPPIS-Finder found covering 22.91% of actually sites in the predicted site. Contrary to this, the sites predicted by SPPIDER covered 22.7% of actual sites. However, on predicting two PPI sites for each protein-chain, the percentage coverage of actual sites in the predicted sites by YAPPIS-Finder exceeded two-fold (i.e. 41.81%), thus making the YAPPIS-Finder a superior approach.

## Conclusion:

This paper describes the development of a novel, multiparameteric-method YAPPIS-Finder to identify the protein-protein interaction sites. The YAPPIS-Finder was tested on a set of above 500 protein-protein complexes. The ability of the method in identifying the protein-protein interaction sites has been investigated. The YAPPIS-Finder method included the information of solvation. Residue propensity, hydrophobicity and van der Waals interaction energy in its ability to identify near-precise region of protein-protein interactions whilst at the same time giving a higher degree of correlation in overlap between predicted and experimentally proved protein-protein interaction sites.
